# Human Heart-Macrophage Assembloids Mimic Immune-Cardiac Interactions and Enable Arrhythmia Modeling

**DOI:** 10.51894/001c.164003

**Published:** 2026-07-31

**Authors:** Colin O’Hern, Sammantha Caywood, Shakhlo Aminova, Artem Kiselev, Brett Volmert, Nagib Chalfoun, Sangbum Park, Nureddin Ashammakhi, Christopher Contag, Aitor Aguirre

**Affiliations:** 1 College of Osteopathic Medicine, Michigan State University

## 07

### Introduction

Yolk-sac–derived embryonic cardiac tissue-resident macrophages (TRMPs) colonize the heart early in development and play indispensable roles in cardiogenesis, including tissue remodeling, angiogenesis, electrical conduction, efferocytosis, and immune regulation. However, mechanistic interrogation of human TRMP–cardiomyocyte interactions has been limited by the lack of physiologically relevant human model systems. Here, we report the development of a human heart–macrophage assembloid (hHMA) by integrating autologous human pluripotent stem cell–derived embryonic monocytes into self-organizing heart organoids, enabling the generation of bona fide TRMP-like macrophages within a human cardiac microenvironment.

### Objectives

We hypothesized that TRMPs contribute to the developmental trajectory of hHMAs and atrial arrhythmias with chronic inflammatory stimulation.

### Methods

We added hPSC-derived monocytes to an hPSC-derived human heart organoid, seeding a physiologically relevant TRMP population in hHMAs. Then, we utilized single-cell RNA sequencing, RT-qPCR, live-cell imaging, and proteomic analyses to characterize the TRMP contribution to hHMA development and homeostasis. Lastly, we chronically exposed hHMAs to pro-inflammatory factors (IL-1, IFN-γ, and LPS) to induce atrial arrhythmias in hHMAs and used functional (FluoVolt live-cell imaging), transcriptomic (RT-qPCR), and proteomic (immunofluorescent microscopy) analyses to characterize the system.

### Results

We demonstrated that TRMPs actively regulate hHMA development by modulating paracrine signaling networks, mediating efferocytosis of apoptotic cells, remodeling the extracellular matrix, and influencing cardiac electrical conduction. Functional perturbation studies reveal that macrophage–cardiomyocyte crosstalk is essential for proper structural and electrophysiological maturation of the hHMAs. Furthermore, in a proof-of-concept matured hHMA model of chronic inflammation, TRMPs undergo phenotypic reprogramming toward a pro-inflammatory state that induces arrhythmogenic activity, consistent with features of atrial fibrillation.

### Conclusions

Together, this hHMA provides a powerful and scalable in vitro platform to dissect immune–cardiac interactions during human heart development and to model inflammation-driven cardiac arrhythmias in a human-relevant context.

